# Retraction Note: A Chinese herbal formula, Jian-Pi-Yi-Shen decoction, improves muscle atrophy via regulating mitochondrial quality control process in 5/6 nephrectomised rats

**DOI:** 10.1038/s41598-021-91869-x

**Published:** 2021-06-14

**Authors:** Dongtao Wang, Jianping Chen, Xinhui Liu, Ping Zheng, Gaofeng Song, Tiegang Yi, Shunmin Li

**Affiliations:** 1 Department of Nephrology, Shenzhen Traditional Chinese Medicine Hospital, Guangzhou University of Chinese Medicine, Shenzhen, 518033 China; 2 Shenzhen Key Laboratory of Hospital Chinese Medicine Preparation, Shenzhen Traditional Chinese Medicine Hospital, Guangzhou University of Chinese Medicine, Shenzhen, 518033 China; 3 Department of Nephrology, Ruikang Affiliated Hospital, Guangxi University of Chinese Medicine, Nanning, 530011 China

Retraction of: *Scientific Reports* 10.1038/s41598-017-10027-4, published online 23 August 2017

The authors have retracted this article.

After publication they became aware of a number of irregularities in the figures, specifically:

- In Figure 3a the Sham band appears to be identical for the Atrogin-1 and MuRF1 blots
- In Figure 1d the Sham and CKD panels appear to partially overlap
- In Figure 4a the Sham and JPYS panels appear to partially overlap

Dongtao Wang, Jianping Chen, Xinhui Liu, and Shunmin Li agree to this retraction. Ping Zheng, Gaofeng Song, and Tiegang Yi did not respond to correspondence about this retraction.

